# Cowden syndrome–like phenotype with lymphangioma circumscriptum and an APC variant of uncertain significance

**DOI:** 10.1016/j.jdcr.2026.06.050

**Published:** 2026-06-27

**Authors:** Fatma Omar Al Hosni, Thuraiya Hilal Al Rumhi

**Affiliations:** a Department of Dermatology, Al Khuwair Dermatology Center, Muscat, Sultanate of Oman; b Department of Dermatology, Medical City of Military and Security Services, Muscat, Sultanate of Oman

**Keywords:** APC mutation, Cowden syndrome, genetic testing, lymphangioma circumscriptum, PTEN-like phenotype, radiation-induced skin changes

## Introduction

Cowden syndrome is a rare genetic disorder characterized by hamartomatous tumor formation and increased risk of malignancies, typically caused by germline pathogenic variants in the phosphatase and tensin homolog deleted on chromosome 10 (PTEN) gene.^1^^,^^2^ Diagnosis is typically based on a combination of clinical criteria and confirmatory genetic testing.^3^ Herein, we present a unique case of a female patient who fulfilled the clinical diagnostic criteria for a Cowden syndrome–like phenotype but was found to carry an adenomatous polyposis coli (APC) variant of uncertain significance rather than the typical PTEN alteration. Despite genetic evaluation, no definitive molecular diagnosis could be established.

Notably, she developed lymphangioma circumscriptum (LC), a rare cutaneous lymphatic malformation, which, to our knowledge, has not been previously reported in association with an APC variant of uncertain significance or in this clinical context.

## Case presentation

A 35-year-old female with a clinical diagnosis of CS-like phenotype presented with multiple characteristic features. Oral examination revealed crowded teeth and cobblestone-appearing gums and tongue (Fig 1). Dermatological assessment showed multiple skin-colored, flat-topped papules on the face clinically consistent with facial trichilemmomas. Additionally, hyperkeratotic papules resembling **acral keratoses** were observed on the dorsal aspects of her hands and feet (Fig 2).

**Fig 1:**
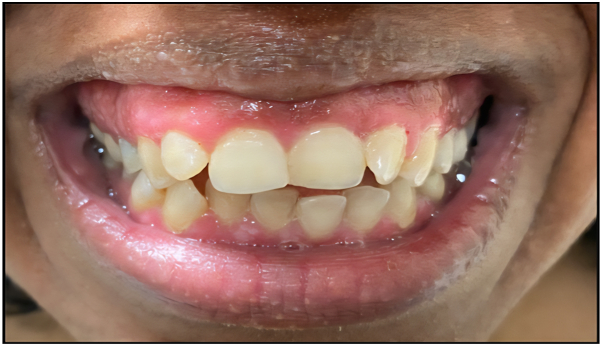
Oral examination shows crowded teeth and cobblestone-appearing gums and tongue in a patient with Cowden-like features.

**Fig 2:**
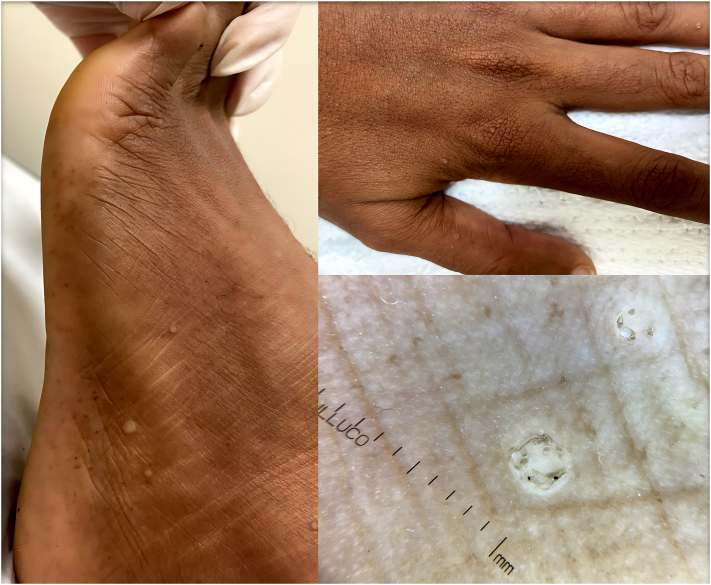
Hyperkeratotic papules on the dorsal aspects of hands and feet resembling punctate palmoplantar keratosis.

Her medical history was significant for multiple benign tumors, beginning at age 13, including nodular colloid goiter, right breast fibroadenoma, angiolipomas of the left foot and kidney, mature cystic teratoma of the left ovary, cystadenoma of the right ovary, endometrial polyps, gastrointestinal polyps, liver hemangioma, and benign lung nodules. A new thyroid nodule on the left side is currently under evaluation. She was also diagnosed with low-grade ductal carcinoma in situ of the left breast, which was estrogen receptor and progesterone receptor positive and HER2-negative, with negative BRCA testing. She underwent wide local excision with sentinel lymph node biopsy followed by adjuvant radiotherapy (total dose 50 Gy delivered in 25 fractions over 2 months).

Family history was notable for **parental consanguinity** (**first cousins**) and multiple relatives with breast, colon, and uterine cancers, as well as thyroid nodules and ovarian dermoid cysts in her mother.

A punch biopsy was taken from the left chest skin lesion. Histopathology revealed ectatic, thin, and anastomosing vascular channels without cytologic or architectural atypia, confirming lymphangioma circumscriptum (Fig 3). The lesion was located in an area previously treated with radiotherapy. The patient underwent several sessions of cryotherapy resulting in lesion resolution with residual hyperpigmentation.

**Fig 3:**
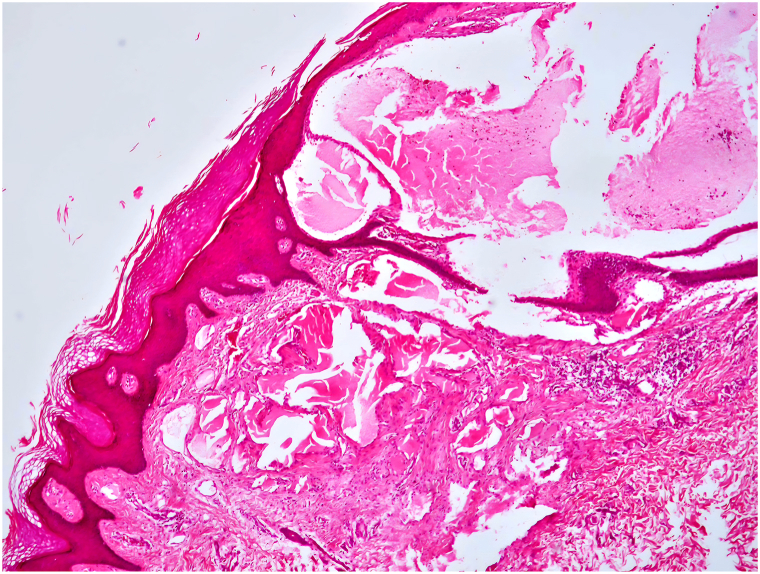
Histopathology of a chest skin lesion reveals ectatic, thin, and anastomosing vascular channels without cytological or architectural atypia, confirming lymphangioma circumscriptum.

Genetic testing using whole-exome sequencing revealed a heterozygous variant of uncertain significance in the APC (c.6639G>A, p.Met2213Ile), with conflicting interpretations of pathogenicity. No pathogenic variant was identified that explains the clinical phenotype, and no definitive genetic diagnosis was established based on the available testing. Exome-wide copy number variation analysis did not detect any clinically significant large deletions or duplications.

She remains under multidisciplinary care, including genetics, gynecology, breast surgery, gastroenterology, surgery, oncology and endocrinology.

## Discussion

CS is a rare autosomal dominant disorder primarily caused by germline pathogenic variants in the PTEN tumor suppressor gene, leading to dysregulated PI3K/AKT signaling and tumorigenesis.^1^^,^^2^ However, variants in other genes, including succinate dehydrogenase complex subunit B, succinate dehydrogenase complex subunit C, and succinate dehydrogenase complex subunit D, have been described in patients with CS-like phenotypes.^4^

Our patient fulfilled the revised clinical diagnostic criteria for PTEN hamartoma tumor syndrome, meeting more than 2 major and 3 minor criteria, as outlined in Table I.^3^ Specifically, she presented with major criteria including breast cancer and gastrointestinal hamartomas, along with acral hyperkeratotic papules, oral papillomas, and trichilemmoma-like lesions (all diagnosed clinically). Additional minor criteria included vascular lesions, thyroid structural lesions, and lipomas. However, genetic testing identified an APC variant of uncertain significance (c.6639G>A, p.Met2213Ile) instead of PTEN. Based on the genetic report, this variant has conflicting interpretations and should not be considered causative. Importantly, no definitive molecular diagnosis was established in this case.

**Table I:** Revised clinical diagnostic criteria for PTEN hamartoma tumor syndrome (PHTS)

Major criteria	Minor criteria
Breast cancer	Colon cancer
Epithelial endometrial cancer	Renal cell carcinoma
Follicular thyroid cancer (nonmedullary)	Papillary or other thyroid cancer
Gastrointestinal hamartomas (≥3)	Thyroid structural lesions (eg, nodules)
Adult-onset Lhermitte–Duclos disease	Esophageal glycogenic acanthosis
Macrocephaly	≥3 lipomas
Macular pigmentation of the glans penis	Vascular anomalies
Multiple mucocutaneous lesions (any of the following)	Mental retardation
≥3 trichilemmomas (at least 1 biopsy-proven)	Testicular lipomatosis
≥3 palmoplantar pits or acral hyperkeratotic papules	Autism spectrum disorder
≥3 mucocutaneous neuromas	
≥3 oral papillomas (biopsy-proven or diagnosed by dermatologist)	

Clinical diagnostic criteria for PHTS.

Diagnosis may be made based on any of the following:

1. Three or more major criteria, with at least 1 being macrocephaly, gastrointestinal hamartomas, or Lhermitte–Duclos disease.

2. Two major criteria and 3 minor criteria.

3. An individual with a first-degree relative with a known PTEN mutation or clinical diagnosis of PHTS, and who meets 1 of the following:

a. Two major criteria.

b. One major and 2 minor criteria.

c. Three minor criteria.

APC pathogenic variants are classically associated with familial adenomatous polyposis and related extracolonic manifestations, including lipomas, fibromas, and epidermal cysts, some of which were also present in our patient.^5^ Although rare, some reports suggest that APC variants may present with extraintestinal findings that overlap with features seen in PTEN syndromes. However, the clinical boundaries between these phenotypes remain poorly defined.^6^

Cutaneous manifestations of CS typically include multiple mucocutaneous lesions such as trichilemmomas, oral papillomas, acral keratoses, and sclerotic fibromas.^3^^,^^7^ In our case, trichilemmomas and oral papillomas were diagnosed clinically because the patient declined biopsy due to concerns about scarring. These lesions often appear prior to malignancy development.^7^ Hyperkeratotic **acral** papules are a recognized feature of CS,^7^ but have not been commonly reported in association with APC variants. Their presence in this patient further supports a CS-like clinical phenotype despite the absence of a confirmed molecular diagnosis.

In addition, the patient exhibited hyperpigmented macules on the palms and soles. While not a classical feature of either CS or APC-associated conditions, their presence may represent a secondary pigmentary response, postinflammatory change, or incidental finding.

Vascular anomalies, including hemangiomas, venous malformations, arteriovenous malformations, and lymphatic malformations, are recognized as minor diagnostic criteria in CS and related PTEN hamartoma tumor syndromes.^3^^,^^7^ This case uniquely highlights the presence of LC, a superficial lymphatic malformation confirmed histologically. LC is usually congenital but can be acquired secondary to trauma, inflammation, infection, malignancy, surgery, or radiotherapy.^8^, ^9^, ^10^ Radiation-induced LC is rare and typically presents as translucent vesicles developing gradually in previously irradiated areas, due to lymphatic obstruction and dermal fibrosis.^10^

Given the rarity of both CS and the absence of a confirmed molecular diagnosis, LC in this patient may represent either a delayed radiation-induced effect or an incidental finding in the context of a Cowden-like clinical phenotype, rather than a direct manifestation of the identified APC variant of uncertain significance. This interpretation underscores the importance of cautiously correlating genetic findings with the overall clinical picture.

## Conflicts of interest

None disclosed.
